# Intervention for Diagnosis of Deep Vein Thrombosis in Acute Stroke Patients: A Hospital-Based Study

**DOI:** 10.1371/journal.pone.0114094

**Published:** 2014-12-02

**Authors:** Mu-Chien Sun, Meng-Shan Li

**Affiliations:** Stroke Center and Department of Neurology, Changhua Christian Hospital, Changhua, Taiwan; Maastricht University Medical Center, Netherlands

## Abstract

**Background:**

Immobile stroke patients are at high risk of deep vein thrombosis (DVT). Demographic studies suggest a low incidence of DVT in Asian patients, but that might be underestimated.

**Objective:**

Intervention by in-hospital case management for diagnosis of DVT in patients with acute stroke.

**Patients and Methods:**

Intervention was defined as: recommendation of D-dimer test for patients who are immobile by day 4 after stroke onset and compression ultrasonography if the level of D-dimer is ≥500 ng/ml. Treating physicians were notified by case managers before they failed to do so for qualified patients. Data of patients hospitalized 12 months before and 8 months after the intervention, including basic demographics, Glasgow Coma Scale score, National Institute of Health Stroke Scale (NIHSS) score, laboratory results, and examination reports, was retrieved from electronic medical records for analysis by code searches for acute stroke.

**Results:**

A total of 2523 patients were identified. 1528 were before and 995 after intervention. More patients after intervention had D-dimer test and ultrasound examination than that before intervention (22.1% vs 8.6%, P<0.001 and 15.1% vs 1.2%, P<0.001, respectively). Ultrasound diagnosis of DVT was significantly more after than before intervention (2.0% vs 0.3%, P<0.001). DVT was 55.7 per 1000 in patients with a NIHSS score≧18. Male sex (Odds ratio 0.33, 95% confidence intervals: 0.11–0.98), NIHSS score (Odds ratio 1.05, 95% confidence intervals: 1.00–1.09), and intervention (Odds ratio 5.39, 95% confidence intervals: 1.88–15.44) were independent predictors of ultrasound diagnosis of DVT.

**Conclusions:**

Intervention by in-hospital case management may be an effective strategy for improvement of under-diagnosis of DVT in acute stroke patients.

## Introduction

Venous thromboembolism (VTE) includes deep vein thrombosis (DVT) and pulmonary embolism and is an important “hospital acquired” and preventable condition. The latter is the third most common cause of death from cardiovascular disease. [1], [2] Although there are differences between DVT and pulmonary embolism, it is considered appropriate to evaluate both asymptomatic and symptomatic DVT when looking at the effectiveness of prophylaxis for VTE. [3] Diagnosis of DVT is dependent on using a validated clinical decision rule, followed by a quantitative D-dimer test. [1], [4] DVT cannot be ruled out by either a D-dimer test or clinical decision rule alone. [4]

Immobile stoke patients are at high risk of VTE. [5] According to the Wells score for DVT, paralytic patients with recently bedridden >3 days are at immediate risk or high risk if adding one more score for limb swelling or edema which is not uncommon in stroke patients. [1] Based on Padua Prediction Score and American College of Chest Physician guideline, all immobile patients with acute stroke are at high risk of VTE (Padua Prediction Score ≧4). [6] Quantitative D-dimer test is thus justified for immobile stoke patients. [1], [4], [6] Imaging procedure, such as compression ultrasonography should be performed to diagnose DVT if the level of D-dimer is above a threshold of 500 ng/ml. [1], [4], [6] This diagnostic process is in concordance with patients' clinical need based on recommended diagnostic algorithm. [1] The morbidity and mortality attributable to VTE after stroke may be to change with an effective strategy of screening for subclinical DVT. [7] Epidemiological studies on Asians show low incidence of VTE, but it might be underestimated. [8] In one published paper, we have demonstrated that intervention by in-hospital case management may be an effective way to improve diagnosis of atrial fibrillation and anticoagulation therapy for eligible stroke patients. [9] The diagnosis of DVT in acute stroke patients may be also to change by this intervention. The aim of this study is to investigate the change of diagnosis of DVT after intervention.

## Materials and Methods

The Institutional Review Board Committee C at Changhua Christian Hospital (CCH) approved this study for human subjects under the waiver of informed consent. Patient records/information was anonymized and de-identified prior to analysis.

**Figure 1:**
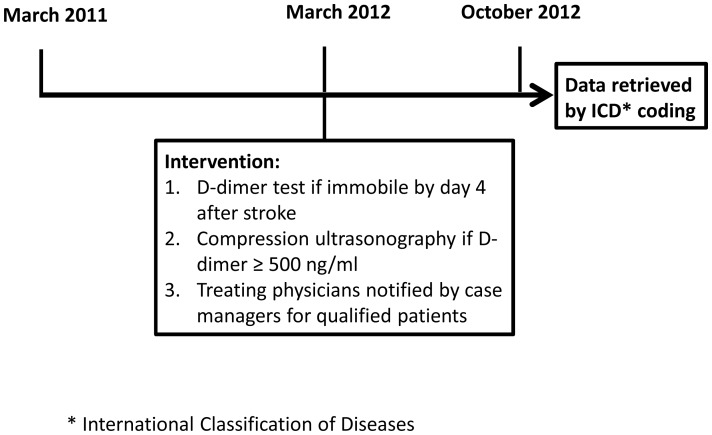
Study profile.

An in-hospital case management for improving quality of stroke care was implemented in the hospital as previous description. [9] In short, in-hospital care process for stroke patients was standardized according to clinical guidelines and monitored by case managers. Because of assumed low VTE incidence in acute stroke patients, pharmacological and mechanical prophylaxes were not used in the hospital. Since March 2012, an intervention for DVT diagnosis by in-hospital case management was implemented in the hospital. (Figure 1) The intervention was defined as: recommendation of D-dimer test for patients who were immobile by day 4 after stroke onset and compression ultrasonography if the level of D-dimer was ≥500 ng/ml. A complete whole leg compression ultrasonography of both legs was performed and reported by hematologists as a routine process in the hospital. Treating physicians were notified by case managers before they failed to do so for qualified patients. However, the intervention was not compulsory.

Data of patients hospitalized between March 2011 and October 2012 including basic demographics, Glasgow Coma Scale score, National Institute of Health Stroke Scale (NIHSS) score, laboratory results, and examination reports was retrieved from electronic medical records for analysis by International Classification of Diseases code searches for stroke. (Figure 1) Beginning with the onset of stroke and the following one month is under a code of “catastrophic illness” for acute stroke defined by the National Health Insurance in Taiwan. Acute stroke patients were further identified by this coding. Patients who were hospitalized between March 2011 and February 2012 were defined as group of “before” intervention. Patients who were hospitalized between March 2012 and October 2012 were defined as group of “after” intervention. Proximal DVT was defined as thrombosis of the popliteal vein or one of the veins above. Severe stroke was defined as a NIHSS score≧18. Mild and moderate stroke were defined as a NIHSS score 0 to 9 and 10 to 17, respectively.

Statistical analyses were performed with SPSS for Windows, version 13.0. Characteristics of patients between “before” and “after” groups were compared statistically. Univariate analysis was carried out using the unpaired t test and χ square test. Independent factors related to ultrasound diagnosis of DVT were analyzed by logistic regression analysis. D-dimer test, compression ultrasonography, and diagnosis of DVT were further analyzed according to stratification of stroke severity. All statistically significant levels were defined as P<0.05.

## Results

A total of 2523 hospitalized patients with acute stroke were identified. 1528 were before and 995 were after the intervention. There was no statistically significant difference in basic demographics, stroke severity, risk factors, and laboratory results between the “before” and “after” groups. (Table 1) More patients in the “after” group had D-dimer test and ultrasound examination than that in the “before” group (22.1% vs 8.6%, P<0.001 and 15.1% vs 1.2%, P<0.001, respectively). Ultrasound diagnosis of DVT was significantly more after than before the intervention (2.0% vs 0.3%, P<0.001). After the intervention, 190 among 220 patients (86.4%) who had D-dimer test had a D-dimer level ≥500 ng/ml. 140 among them (73.7%) had complete whole leg compression ultrasonography. 18 (12.9%) had ultrasound diagnosis of DVT. 12 in the 18 patients had proximal DVT. Median days to check D-dimer and to perform compression ultrasonography were 4 and 6 after stroke onset, respectively.

**Table 1:** Baseline characteristics of the study patients.

Characteristics	All (n = 2523)	Before intervention (n = 1528)	After intervention (n = 995)	P value
Age, years, mean (SD)	68.2 (13.9)	68.4 (13.7)	67.9 (14.2)	0.427
Male sex, n (%)	1446 (57)	859 (56)	587 (59)	0.168
Body Mass Index	24.1 (5.7)	24.1 (6.6)	24 (4.1)	0.605
GCS, mean (SD)	11.6 (5.2)	11.5 (5.2)	11.6 (5.1)	0.710
NIHSS, mean (SD)	12.0 (11.6)	12.1 (12.0)	11.9 (11.0)	0.688
Laboratory Results, mean (SD)				
GPT, U/L	28.7 (50.6)	28.6 (56.3)	28.9 (40.4)	0.902
Hemocrit, %	38.3 (6.4)	38.1 (6.3)	38.5 (6.5)	0.096
Platelet count, 10^3^/µL	221 (98)	221 (102)	220 (92)	0.940
INR	1.1 (0.4)	1.1 (0.5)	1.1 (0.3)	0.487
Past Medical History, n (%)				
Hypertension	1421 (56)	876 (57)	545 (55)	0.206
Diabetes	765 (30)	468 (31)	297 (30)	0.677
Dyslipidemia	734 (29)	428 (28)	306 (31)	0.138
D-dimer Test, n (%)	351 (13.7)	131 (8.6)	220 (22.1)	<0.001
Ultrasonography, n (%)	169 (6.6)	19 (1.2)	150 (15.1)	<0.001
Deep Vein Thrombosis, n (%)	25 (1.0)	5 (0.3)	20 (2.0)	<0.001

GCS, Glasgow Coma Scale; NIHSS, National Institute of Health Stroke Scale; SD, standard deviation.

Table 2 shows the results of multivariate analysis of independent variables related to ultrasound diagnosis of DVT. Male sex (Odds ratio 0.33, 95% confidence intervals: 0.11–0.98), NIHSS score (Odds ratio 1.05, 95% confidence intervals: 1.00–1.09), and the intervention (Odds ratio 5.39, 95% confidence intervals: 1.88–15.44) were independent predictors of ultrasound diagnosis of DVT. Age, Body Mass Index, and platelet count were not related to ultrasound diagnosis of DVT in our analysis.

**Table 2:** Predictors of ultrasound diagnosis of deep vein thrombosis: logistic regression analysis.

Variable	Odds ratio	95% confidence intervals	P value
Age	1.03	0.99–1.08	0.122
Male Sex	0.33	0.11–0.98	0.045
Body Mass Index	1.02	0.91–1.13	0.781
NIHSS	1.05	1.00–1.09	0.034
GPT	0.96	0.91–1.01	0.107
Platelet count	1.00	0.99–1.00	0.557
INR	1.81	0.97–3.36	0.061
Hypertension	1.36	0.49–3.75	0.555
Diabetes	0.92	0.33–2.56	0.867
Dyslipidemia	0.98	0.32–3.03	0.97
Intervention	5.39	1.88–15.44	0.002

NIHSS, National Institute of Health Stroke Scale.

Table 3 shows comparisons for D-dimer test, compression ultrasonography, or ultrasound diagnosis of deep vein thrombosis between patients who were admitted before and after intervention according to stroke severity. There were significantly more D-dimer test and compression ultrasonography for patients with severe stroke after the intervention. Ultrasound diagnosis of DVT was significantly more after than before intervention (55.7 vs 7.5 per 1000, P<0.001) in patients with severe, but not moderate or mild stroke.

**Table 3:** Comparisons for D-dimer test, compression ultrasonography, or ultrasound diagnosis of deep vein thrombosis between patients who were admitted before and after intervention according to stroke severity.

NIHSS	Intervention	N	D-dimer test (%)	Compression ultrasonography (%)	Diagnosis of DVT (per 1000)
18	Before	401	60 (15.0)	11 (2.7)	3 (7.5)
	After	287	107 (37.3)**	111 (38.7)**	16 (55.7)**
10–17	Before	199	16 (8.0)	3 (1.5)	1 (5.0)
	After	142	43 (30.3)**	29 (20.4)**	3 (21.1)
0–9	Before	839	54 (6.4)	5 (0.6)	1 (1.2)
	After	551	70 (12.7)**	10 (1.8)*	1 (1.8)
Missing	Before	89	9 (10.1)	0	0
	After	9	2 (22.2)	0	0

* P<0.05; ** P<0.001; DVT: deep vein thrombosis; NIHSS, National Institute of Health Stroke Scale.

## Discussion

Similar to patients with acute stroke, patients admitted for knee surgery are at high risk of VTE. A study based on National Health Insurance database in Taiwan reported a low incidence (0.07%) of pulmonary embolism within 28 days after knee replacement. [10] The result was lower than that (0.28%) reported from a hospital-based study on symptomatic pulmonary embolism within 8 days after total knee arthroplasty. [11] A multinational Asian study based on postoperative screening reported a VTE incidence that is comparable to Western patients. [12] Thus, low incidence of VTE in Asians based on epidemiological studies may be underestimated.

A low symptomatic DVT rate (0.2%) was found in the Taiwan Stroke Registry. [13] It was suggested that DVT is rarely symptomatic among Asians. Incidence of ultrasound diagnosis of DVT before intervention in our analysis was similar to the rate. It is considered appropriate to evaluate both asymptomatic and symptomatic DVT in terms of VTE prophylaxis. [3] Our results indicate that without intervention DVT is significantly underdiagnosed and thus may not justify VTE prophylaxis.

It is arguable to routinely use VTE prophylaxis for hospitalized patients. [14] However, guidelines strongly recommend VTE prophylaxis for patients at high risk. [3], [5], [6], [13], [14] Immobile stroke patients are among patients at high risk of VTE. High risk patients defined by the Padua Prediction Score ≥4 have 11.0% of VTE risk per year, including 6.7% DVT, 3.9% nonfatal pulmonary embolism, and 0.4% fatal pulmonary embolism. [5] In our analysis, 190 in 220 (86.4%) patients with D-dimer test had a D-dimer level ≥500 ng/ml. 18 in the 190 patients (9.5%) had ultrasound diagnosis of DVT. According to analysis based on stratification of stroke severity, ultrasound diagnosis of DVT in severe stroke patients was 5.6% after intervention. The results of our analysis support that immobile stroke patients are at high risk of DVT.

We retrieved the data by code searches. Coding errors may be a limitation of our study. Because in-hospital case management at CCH monitors both coding for stroke and “catastrophic illness”, we believe that coding errors of these two conditions are few if any. We did not analyze breaching of the intervention protocol due to data limitation. This might introduce selection bias. However, the percentage of DVT diagnosis would only increase by reducing protocol breaching because of a fixed total patient number. The other limitation of our study is that management, the direct consequence of the intervention, for diagnosed patients was not analyzed. The management of diagnosed DVT was left to responsible physicians. Further investigation, including using an age-dependent D-dimer cut-off value, is warranted to evaluate the cost-effectiveness of this strategy to identify high risk patients for VTE prophylaxis. [15]

The results of our study demonstrate that DVT is under-diagnosed in acute stroke patients and intervention by in-hospital case management may be an effective strategy for improvement.

## Supporting Information

Data S1 **Data file (in SPSS, Statistical Package for the Social Sciences).**
(SAV)
